# Unraveling the Interaction between Inflammation and the Cardiometabolic Index in Older Men: A Pilot Study

**DOI:** 10.3390/nu16152529

**Published:** 2024-08-02

**Authors:** Rafael L. Carvalho, Tábatta R. P. Brito, Jônatas B. Amaral, Fernanda R. Monteiro, Daniela B. Lima, Thalles A. M. Pereira, Beatriz F. da Costa, Guilherme E. Furtado, Pamella M. M. Rodrigues, Carlos A. F. dos Santos, André L. L. Bachi, Adriana de Oliveira Sarmento

**Affiliations:** 1 Postgraduate Program in Biomedical Engineering, Anhembi Morumbi University, Technological Innovation Park, São José dos Campos 12247-016, SP, Brazil; rafaaellcarvalho3@gmail.com (R.L.C.); adriana.sarmento@alumni.usp.br (A.d.O.S.); 2 Faculty of Nutrition, Federal University of Alfenas, Alfenas 37130-001, MG, Brazil; tabatta.brito@unifal-mg.edu.br (T.R.P.B.); daniela.lima@unifal-mg.edu.br (D.B.L.); thalles.marques@sou.unifal-mg.edu.br (T.A.M.P.); 3 ENT Research Laboratory, Department of Otorhinolaryngology—Head and Neck Surgery, Federal University of Sao Paulo (UNIFESP), São Paulo 04021-001, SP, Brazil; jbamaral@unifesp.br; 4 Postgraduate Program in Health Sciences, Santo Amaro University (UNISA), São Paulo 04829-300, SP, Brazil; monteiro.fernanda13@gmail.com; 5 Faculty of Pharmacy, Campus Interlagos, Santo Amaro University, São Paulo 04829-300, SP, Brazil; beatrizfalconcosta@gmail.com; 6 Polytechnic University of Coimbra, Rua da Misericórdia, Lagar dos Cortiços, S. Martinho do Bispo, 3045-093 Coimbra, Portugal; guilherme.furtado@ipc.pt; 7 SPRINT—Sport Physical Activity and Health Research & Innovation Center, Polytechnic Institute of Coimbra, 3030-329 Coimbra, Portugal; 8 Research Centre for Natural Resources Environment and Society (CERNAS), Polytechnic Institute of Coimbra, Bencanta, 3045-601 Coimbra, Portugal; 9 Brazilian Institute of Teaching and Research in Pulmonary and Exercise Immunology (IBEPIPE), São Paulo 12245-520, SP, Brazil; pamella.ssmiranda@gmail.com; 10 Postgraduate Program in Translational Medicine, Department of Medicine, Paulista School of Medicine, Federal University of São Paulo (UNIFESP), São Paulo 04023-062, SP, Brazil; carlos.freitas@unifesp.br; 11 Discipline of Geriatrics and Gerontology, Department of Medicine, Paulista School of Medicine, Federal University of Sao Paulo (UNIFESP), São Paulo 04020-050, SP, Brazil

**Keywords:** inflammation, aging, obesity, inflammaging, metaflammation, cardiometabolic index, IL-10, inflammatory cytokine

## Abstract

Both cardiometabolic and chronic inflammatory diseases pose a significant challenge to global public health, particularly among older adults. Here, we investigated the interplay between systemic inflammatory status and the cardiometabolic index (CMI) in older men with adequate weight or obesity. In this observational cross-sectional study, older men (71.79 ± 7.35 years) were separated into groups with normal weight (NW, *n* = 34) and obesity (O, n = 32) to assess circulating levels of pro- and anti-inflammatory cytokines and CMI. Overall, the O group showed not only a higher inflammatory status but also increased CMI (*p* < 0.0001) compared with the NW group. Interestingly, only positive correlations were found between pro- and anti-inflammatory cytokines in both groups. Through multivariate regression analysis, IL-6 (β = −0.2276, *p* = 0.0003) and IL-10 (β = 0.2023, *p* = 0.0030) significantly influenced CMI in the NW group. No significant results were found in the O group. Our findings reinforce the effects of obesity in inflammaging, as well as suggesting that the influence of cytokines in CMI occurs in older men with normal weight, since the elevated pro-inflammatory profile observed in older men with obesity can interfere in this effect.

## 1. Introduction

Chronic inflammatory diseases pose a significant challenge to global public health, especially among older adults, since it is known that the pace at which the population is aging has accelerated. In fact, there is an estimate that the population over 60 years of age will increase from 12% to 22% between 2015 and 2050 [1]. Diseases such as atherosclerosis, Type 2 diabetes, rheumatoid arthritis, and various neurodegenerative diseases are often mediated by chronic inflammatory processes. For instance, the global prevalence of Type 2 diabetes is 10.5% among the adult/older populations (20–79 years), and the total number of people living with diabetes is projected to rise to 643 million by 2030 [2,3].

The World Health Organization (WHO) estimates that 1.28 billion adults aged 30 to 79 have hypertension, with the majority living in low- and middle-income countries. This significant increase is attributed to population growth and aging, as the prevalence of hypertension increases with age, being a significant risk factor for cardiovascular and renal diseases [4,5]. Epidemiological data indicate that chronic inflammation underlying these conditions is a major contributor to morbidity and mortality in older adults, affecting quality of life and increasing the economic burden on healthcare systems [6].

Systemic, sterile, low-grade chronic inflammation associated with aging, also known as “inflammaging”, is characterized by a systemic pro-inflammatory status. This persistent inflammatory state, although less intense than acute inflammation, is associated with various physiological and pathological alterations. As age advances, the ability to resolve inflammatory processes diminishes, leading to several outcomes, including immune dysfunction, which contributes to the development and progression of several chronic diseases. Studies have shown that elevated inflammatory markers, especially cytokines, in older adults are correlated with an increased risk of cardiovascular events, cognitive decline, and mortality [6,7].

Beyond aging, obesity is another pillar in promoting a state of low-grade chronic inflammation. In particular, visceral adipose tissue acts as an active endocrine organ, secreting diverse pro-inflammatory cytokines and adipokines. This subclinical inflammatory state is closely associated with insulin resistance, dyslipidemia, and hypertension, all components of metabolic syndrome. Chronic inflammation induced by obesity not only affects metabolism but also exacerbates inflammaging, amplifying its negative health effects [8,9].

It is broadly accepted that measuring circulating inflammatory cytokines with both pro- and anti-inflammatory properties is useful for assessing the systemic inflammatory status of people in healthy or unhealthy conditions. For instance, cytokines such as interleukins IL-1β, IL-6, IL-8, tumor necrosis factor-alpha (TNF-α), and interferon-gamma (IFN-γ) are well-known pro-inflammatory molecules and key markers involved in the development of both chronic inflammatory and age-related diseases, as well as inflammaging [10,11]. 

In contrast, the cytokine IL-10 presents an anti-inflammatory activity that helps counterbalance pro-inflammatory effects; thus, its presence indicates that the body has activated a pivotal regulatory mechanism in order to maintain homeostasis [12]. According to this information, the assessment of cytokines, along with some other inflammatory-related molecules, not only allows for improved understanding concerning the systemic inflammatory status but may also have a potential impact on cardiometabolic health in different populations, particularly in older adults presenting with a normal weight or obesity.

In this context, the cardiometabolic index (CMI) emerges as a relevant predictive marker for diagnoses associated with inflammatory cardiovascular diseases [13,14]. The CMI integrates metabolic variables, such as triglyceride and HDL cholesterol (HDL-c) levels, and anthropometric data (waist circumference and height), providing a robust measure of cardiometabolic risk [15]. Studies have demonstrated that an elevated CMI is associated with poorer prognosis in chronic inflammatory conditions, reflecting the combined impact of metabolic and inflammatory factors on cardiovascular health [13,16]. 

Although the literature highlights a close interplay between inflammaging and obesity, the overlapping effects of these conditions in CMI in the aged population are not fully understood. Therefore, the current pilot study aimed not only to assess the systemic inflammatory profile in older adults presenting with adequate weight and obesity, but mainly to investigate whether there is a connection between the inflammatory profile with CMI in these volunteer groups. Understanding these interactions could provide valuable insights for preventive and therapeutic interventions, aiming to improve the health and well-being of older individuals at risk for chronic inflammatory diseases.

## 2. Materials and Methods

### 2.1. Study Design

This was an observational cross-sectional pilot study involving older men aged between 65 and 85 years, separated into two groups, based on the body mass index: adequate (normal) weight (NW) and obesity (O). It is worth mentioning that the study’s primary endpoint was to investigate the possible (in)direct association of the inflammatory profiles present in the volunteer groups with the cardiometabolic index (CMI). Additionally, the study followed the Strengthening the Reporting of Observational Studies in Epidemiology (STROBE) guidelines to ensure methodological rigor and transparent reporting [17].

### 2.2. Ethical Statement

After the initial invitation, all participants were informed about the research’s objectives as well as the confidentiality of the data. Volunteers who agreed to participate were asked to sign the informed consent form, previously approved by the Research Ethics Committee of the Federal University of Alfenas (UNIFAL under number 85218518.0.0000.5142). The present study respected the Brazilian Resolution (196/96) on ethics in research with humans [18], and is in agreement with the guidelines of the Helsinki Declaration.

### 2.3. AI Assistance in Manuscript Preparation

Artificial intelligence tools were utilized in this study exclusively for editing, translation, and proofreading purposes, with no authorship role attributed to the software. The following AI tools were used.

Consensus.app (available at https://consensus.app/search/, Free version, accessed on May 2024). This tool was used to assist in the search and compilation of relevant bibliographic references.

Grammarly, developed by Grammarly Inc., San Francisco, CA, USA (available at https://www.grammarly.com/, Free version, accessed on May 2024): This tool provided assistance in writing, translation, and proofreading of the manuscript.

All content generated or assisted by these AI tools was meticulously reviewed and edited by the authors to ensure its originality, validity, and compliance with MDPI’s policies on publication ethics.

### 2.4. Participants’ Selection Criteria

To perform this pilot study, the following inclusion criteria were applied: older men aged between 60 and 89 years who agreed to participate in the study spontaneously, had autonomy to move from their residence to the site of data collection and sampling, and had a BMI ranging between 18 and 24.9 for the normal weight group and between 30 and 39.9 for the obese group. In addition, the following exclusion criteria were established: the presence of undernutrition, overweight, and Grade III obesity; senile dementia; seropositivity for HIV infection; physical illness and/or acute or chronic infections; liver and renal diseases; and the use of anti-inflammatory drugs during the month prior to collection of the sample.

In addition, the presence of some common aging-related comorbidities (hypertension, Type 2 diabetes, cardiovascular diseases, stroke, and cancer), was considered, and a physical activity questionnaire was applied to ensure homogeneity between the groups. It is also important to clarify that the definitions of both the BMI classifications and levels of physical activity were in agreement with the WHO’s guidelines.

### 2.5. Calculation of the Sample Size 

**Figure 1:**
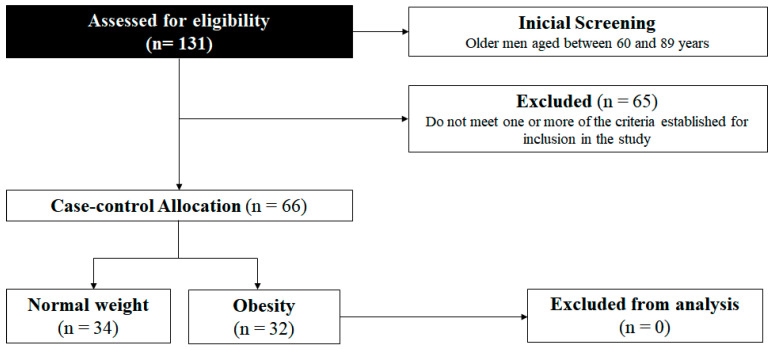
Flowchart of the study design.

In order to ensure the representativeness of the groups, the sample size was calculated using the G*Power software program version 3 [19], and standard parameters for an independent sample *t*-test, considering a medium effect size (0.3) at a significance α-level of 0.05 and a statistical power of 0.80. Considering 30% losses or refusals [18], this calculation indicated that a minimum of 32 participants per group would be necessary. As shown in the flowchart (Figure 1), 131 older men from the community were initially invited to participate in the present study. Taking the previously determined participant selection criteria into account, 65 volunteers were excluded and 66 were allocated into the two groups, according to their BMI values: normal weight (n = 34) and obese (n = 32).

### 2.6. Clinical and Physical Data

Initially, all anthropometric measurements were taken, and information regarding health and physical activity was collected through questionnaires, which were administered during a 1-h interview. Measurements of height were taken using a stadiometer and weight in kilograms was taken using a digital scale, following the standard protocol, with participants wearing light clothing and barefoot. The BMI was obtained using the formula BMI = weight (kg)/height (m²). The classification of participants as normal weight, overweight, and obese followed the criteria proposed by the World Health Organization (WHO). According to these criteria, normal weight was defined as a BMI of 18.5 to 24.9 kg/m², overweight as a BMI of 25 to 29.9 kg/m², obesity Class I as a BMI of 30 to 34.9 kg/m², and obesity Class II as a BMI of 35 to 39.9 kg/m² (Source: WHO, 2023 [1]). 

The waist circumference measurement was conducted using a non-elastic measuring tape. Participants were instructed to stand upright with their feet together, ensuring that their weight was evenly distributed on both legs, and with their arms relaxed at their sides. The tape was positioned horizontally around the abdomen at the midpoint between the last rib and the top of the iliac crest. The tape was adjusted to be snug but not tight, without compressing the skin. Measurements were taken to the nearest 0.1 cm, ensuring that the tape was parallel to the floor. Care was taken to avoid any clothing that could interfere with the accuracy of the measurement. This procedure was conducted in a private setting to ensure the participants’ comfort and privacy, following standardized anthropometric measurement protocols.

The interviewer collected self-reported information from participants regarding the presence of disorders such as Type 2 diabetes and hypertension, as well as their weekly physical activity practices, including light walking, light jogging, gymnastics, weight training, and swimming. Based on the responses to the questionnaire, we calculated the weekly level of physical activity according to the WHO’s guidelines [20], thereby enabling the classification of participants as either physically active or inactive.

### 2.7. Blood Sample Collection

In a second phase, after the collection of clinical and anthropometric data, fasting blood samples were collected in tubes containing the anticoagulant EDTA. Aliquots of the plasma obtained after centrifugation (800× *g* for 10 min) were maintained at −80 °C until their use for analyses of the lipid profile and inflammatory markers.

### 2.8. Analysis of the Lipid Profile 

Circulating levels of high-density lipoprotein cholesterol (HDL-c) and triglycerides were determined using colorimetric commercial kits (Labtest Diagnóstica, Minas Gerais, Brazil), and the results were analyzed in the Thermo Scientific Multiskan SkyHigh microplate spectrophotometer (ThermoFisher, Waltham, MA, USA).

### 2.9. Cardiometabolic Index

To calculate the cardiac metabolic index (CMI), the following formula was used: CMI = (TG/HDL-c) × WHtR, where TG represents triglycerides, HDL-c stands for high-density lipoprotein cholesterol, and WHtR is the waist circumference to height ratio. Both waist circumference and height were measured in centimeters. This formula integrates lipid levels and visceral adiposity, providing a comprehensive indicator of cardiometabolic risk. The values of triglycerides and HDL-c were obtained from fasting blood samples, while waist circumference and height were measured using standard anthropometric procedures. This calculation enabled the assessment of each individual’s risk of cardiometabolic conditions, considering both biochemical and physical parameters [16].

### 2.10. Multiplex Cytokine Analysis 

Plasma cytokine concentrations of IL-1β, IL-6, IL-8, IL-12p70, IP-10, GM-CSF, TNF-α, IFN-γ, and IL-10 were determined using the LEGENDplex Human Custom Cytokine Panel (BioLegend, San Diego, CA, USA). All samples were measured in duplicate to ensure accuracy and reproducibility. Briefly, frozen plasma samples were first thawed on ice and mixed with a solution containing specific beads provided by the manufacturer. After 2 h, the samples were washed and incubated with detection antibodies at room temperature for 1 h, followed by incubation with streptavidin–phycoerythrin (PE) for 30 min while protected from light. Subsequently, the samples were submitted to a final wash, and data acquisition was performed using a BD Accuri™C6 flow cytometer (BD Biosciences, San Jose, CA, USA). For each sample, more than 300 beads were recorded and analyzed using the Data Analysis Software Suite for LEGENDplex 8.0, a free cloud-based program available at the LEGENDplex Analysis Suite. The assay linearity for each parameter was within the 2.4–10,000 pg/mL range, the correlation coefficients of all standard curves were between 0.94 and 0.99, the intra-assay coefficients of variance were 3–8%, and the inter-assay coefficients of variance were 7–10%.

### 2.11. Statistical Analysis

Initially, all data obtained were assessed for normality and homogeneity of variance using the Shapiro–Wilk and Levene tests, respectively. Based on the results obtained, comparisons between the two independent groups were performed using either the unpaired Student’s *t*-test for parametric data or the Mann–Whitney U-test for non-parametric data. For the analysis of qualitative data in contingency tables, Fisher’s exact test was used. In order to verify the occurrence of correlations among the studied variables, Spearman’s rank correlation coefficient test was applied, enabling the construction of a correlation network. Furthermore, a multivariate regression analysis adjusted for CMI was conducted to more comprehensively explore the associations among the variables. We used the backward elimination method for selection of the variables in the multiple linear regression model. In this approach, new models were created by removing the least significant variable in each interaction until only variables with *p* < 0.15 remained in the model. This method is well supported in the statistical literature for its effectiveness in model refinement and improving the interpretability of regression models [21,22]. 

The α risk was set to 5% (*p* < 0.05) for univariate analyses, as each cytokine concentration and calculated ratio was compared separately and individually. To mitigate the risk of Type I errors from multiple comparisons in multivariate analyses, including the cytokine correlation network and the multiple linear regression adjusted for CMI, the α risk was adjusted to 1% (*p* < 0.01). All statistical analyses were performed using GraphPad Prism^®^ version 8.4.3.

## 3. Results

### 3.1. Characterization of the Sample 

Table 1 presents the demographics, anthropometric details, clinical details, and levels of physical activity of each volunteer group. As expected, older adults with obesity presented with a higher weight, BMI, waist circumference, and triglyceride levels, as well as a higher number of individuals with hypertension and lower HDL-c levels than the values observed in older adults with adequate weight. Interestingly, no differences were found in the number of volunteers with Type 2 diabetes and levels of physical activity between the groups.

### 3.2. Analysis of the Cardiometabolic Index 

**Figure 2:**
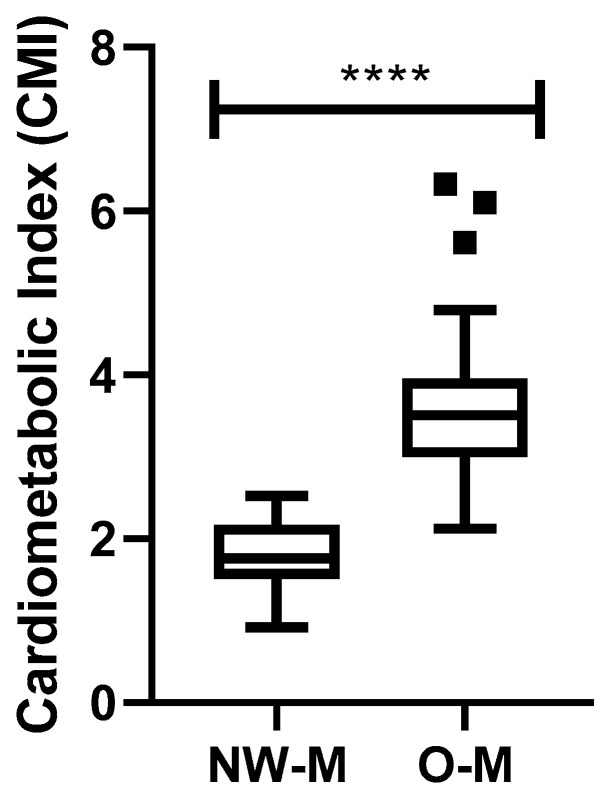
Cardiometabolic index (CMI) in older men with normal weight (NW) and obesity (O). The CMI values were significantly higher in the O group compared with the NW group. The statistical analysis indicated a highly significant difference between the groups (**** *p* < 0.0001).

As shown in Figure 2, the volunteers with obesity (O) presented increased CMI (*p* < 0.0001) compared with the volunteers with an adequate (normal) weight (NW). In addition, the NW group exhibited CMI values ranging from 0.9186 to 2.522, with a median of 1.766 (CI: 1.623–1.937) and a coefficient of variation of 25.27%, while the O group presented CMI values ranging from 2.125 to 6.327, with a median of 3.508 (CI: 3.296–4.047) and a coefficient of variation of 27.39%. 

### 3.3. Analysis of Systemic Cytokines 

**Figure 3:**
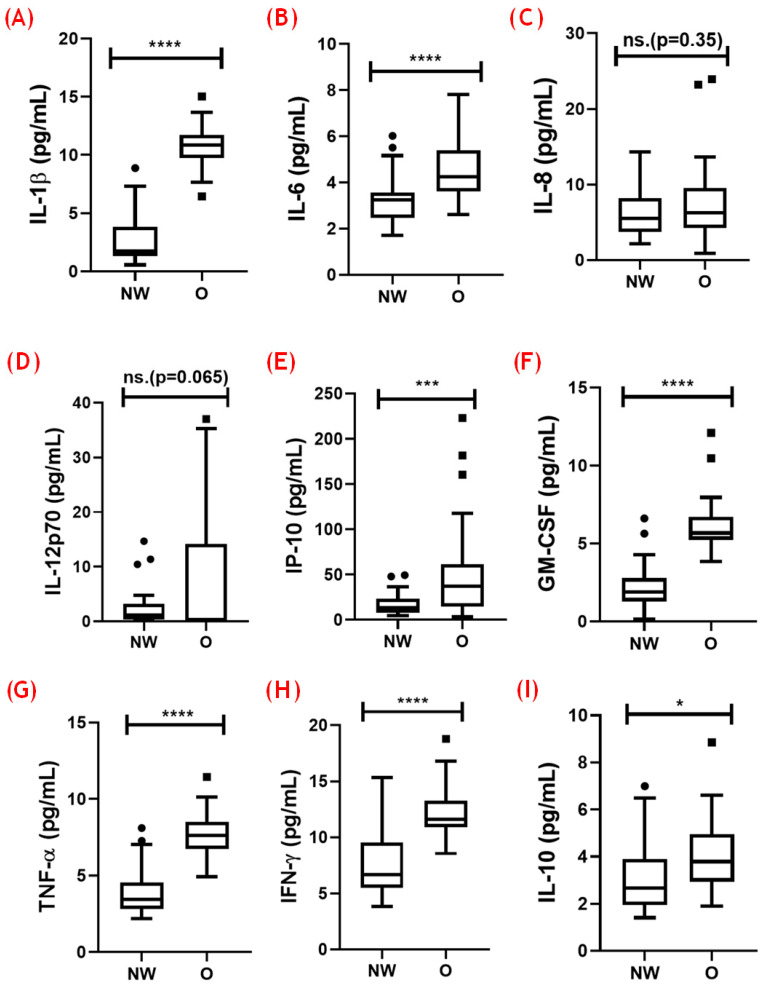
Levels of inflammatory cytokines in older men with normal weight (NW) and obesity (O). The levels of various cytokines were measured using the LEGENDPlex™ (Custom Human Panel 13-plex) from BioLegend. (**A**) IL-1β, (**B**) IL-6, (**C**) IL-8, (**D**) IL-12p70, (**E**) IP-10, (**F**) GM-CSF, (**G**) TNF-α, (**H**) IFN-γ, and (**I**) IL-10. The levels of cytokines are presented in pg/mL. Statistical significance is indicated as follows: * *p* < 0.05; *** *p* < 0.001; **** *p* < 0.0001.

Figure 3 presents the results concerning circulating levels of both pro- and anti-inflammatory cytokines in the volunteer groups. Higher levels of IL-1β (A, *p* < 0.0001), IL-6 (B, *p* < 0.0001), IP-10 (E, *p* < 0.001), GM-CSF (F, *p* < 0.0001), TNF-α (G, *p* < 0.0001), and IFN-γ (H, *p* < 0.0001), and lower IL-10 levels (I, *p* < 0.05) were found in the volunteers with obesity compared with the NW group. No significant differences were found in the systemic levels of IL-8 (C, *p* = 0.35) and IL-12p70 (D, *p* = 0.065) between the volunteer groups.

### 3.4. Analysis of the Balance between Pro- and Anti-Inflammatory Cytokines 

**Figure 4:**
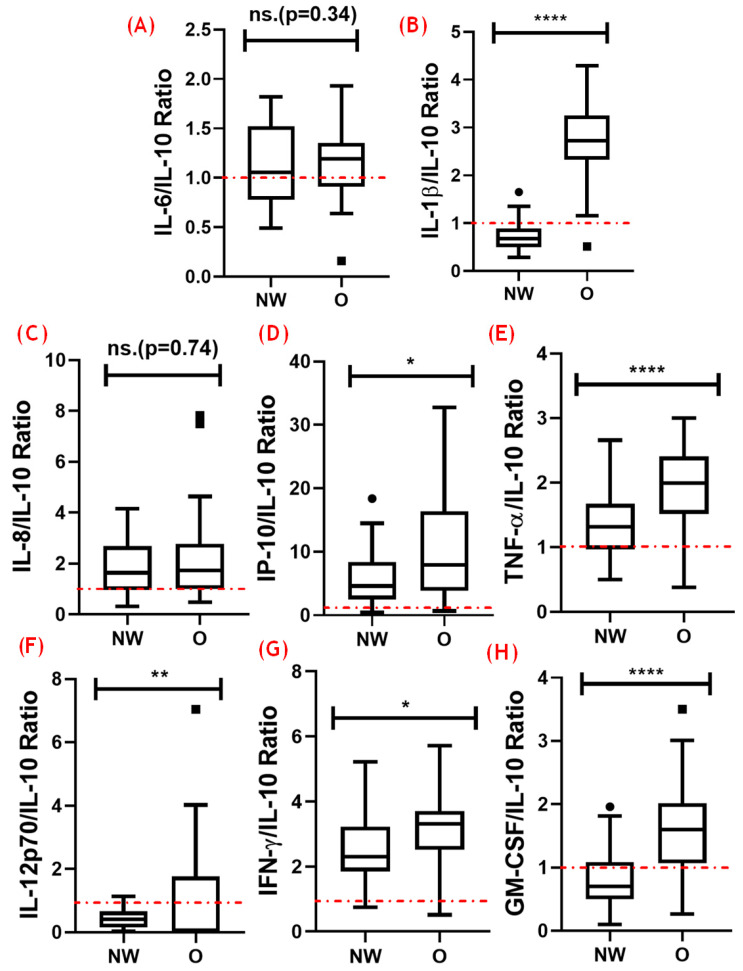
Ratios of pro-inflammatory cytokines to IL-10 in older men with a normal weight (NW) and obesity (O). The ratios of various pro-inflammatory cytokines to the anti-inflammatory cytokine IL-10 were measured. (**A**) IL-6–IL-10, (**B**) IL-1β–IL-10, (**C**) IL-8–IL-10, (**D**) IP-10–IL-10, (**E**) TNF-α–IL-10, (**F**) IL-12p70–IL-10, (**G**) IFN-γ–IL-10, and (**H**) GM-CSF–IL-10. The dashed red line in each panel indicates the baseline ratio for comparison. Statistical significance is indicated as follows: * *p* < 0.05; ** *p* < 0.01; **** *p* < 0.0001; ns, not significant.

Figure 4 presents the results obtained in the analysis of the ratio between the pro-inflammatory cytokines assessed here and the anti-inflammatory cytokine IL-10. The dashed red line shows the value of equality in the ratio between the pro- and anti-inflammatory cytokines. On this basis, it was found that the overwhelming majority of the ratios were above 1, which indicated the presence of a pro-inflammatory status in the volunteer groups. In addition, the volunteers with obesity showed higher ratios between IL-1β and IL-10 (A, *p* < 0.0001), IP-10 and IL-10 (D, *p* < 0.05), TNF-α and IL-10 (E, *p* < 0.0001), IL-12p70 and IL-10 (F, *p* < 0.01), IFN-γ and IL-10 (G, *p* < 0.05), and GM-CSF and IL-10 (H, *p* < 0.0001) than the NW group. No significant differences were observed in the IL-6–IL-10 (B, *p* = 0.34) and IL-8–IL-10 (C, *p* = 0.74) ratios between the groups.

### 3.5. Correlation Analysis of Systemic Inflammatory Cytokines

**Figure 5:**
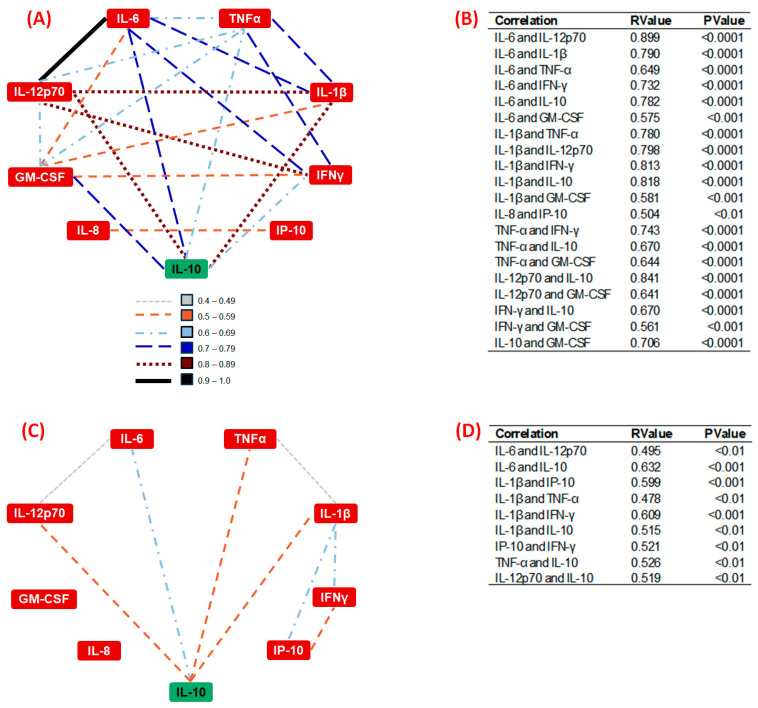
Cytokines’ correlation networks in older men with a normal weight (NW) and with obesity (O). Spearman’s correlation networks among cytokines in (**A**) the NW group and (**C**) the O group, illustrating the relationships among different cytokines. Only positive correlations were observed. The strength of the correlations is indicated by the thickness and color of the lines: gray dashed lines represent correlations with R values of 0.40–0.49, orange dashed lines represent correlations with R values of 0.50–0.59, light blue dashed lines represent correlations with R values of 0.60–0.69, dark blue dashed lines represent correlations with R values of 0.70–0.79, brown dashed lines represent correlations with R values of 0.80–0.89, and black solid lines represent correlations with R values of 0.90–1.0. All correlations shown are statistically significant (*p* < 0.01). Cytokines’ correlations in (**B**) the NW group and (**D**) the O group. This table presents the significant correlations among cytokines in older men with a normal weight (NW) group and obesity (O). The strength of each correlation is represented by the R value, while the *p* value indicates the statistical significance. Only correlations with *p* < 0.01 are included.

Figure 5A,B present the significant correlations among the circulating inflammatory cytokines assessed here in the group of older men with adequate (normal)-weight (NW). Figure 5C,D show the significant correlations found in the group of older volunteers with obesity (O). Specifically, in Figure 5, it was demonstrated that the denser connections in the NW group contrast with the sparser connections in the O group, clearly illustrating the variations in the cytokines’ interaction patterns. In general, the NW group exhibited a denser and more interconnected network of cytokine correlations, which could indicate a more complex and potentially balanced inflammatory state, since this group showed numerous strong correlations, particularly with the anti-inflammatory cytokine IL-10, while the group with obesity showed fewer and weaker correlations.

### 3.6. Analysis of the Interplay between Inflammatory Status and the Cardiometabolic Index

To investigate the potential direct influence of the inflammatory profile on the cardiometabolic index (CMI), a multivariate regression analysis was performed with CMI as the dependent variable.

#### 3.6.1. Results in the NW Group

The results of the multivariate regression analysis in the NW group showed that IL-6 (β = −0.2276; 95% CI −0.3389 to −0.1164; *p* = 0.0003) and IL-10 (β = 0.2023; 95% CI 0.07484 to 0.3297; *p* = 0.0030) had a significant impact on CMI. The regression model demonstrated an adequate fit to the data, with an R² of 0.6443 and a significant F value (F(5, 28) = 10.14, *p* < 0.0001). The other cytokines did not show a significant influence.

#### 3.6.2. Results in the O Group

Concerning the O group, the multivariate regression analysis showed that none of the evaluated cytokines had a significant impact on CMI. The estimated parameters were TNF-α (β = 0.1367; 95% CI −0.02922 to 0.3026; *p* = 0.1025) and IFN-γ (β = 0.1369; 95% CI −0.01033 to 0.2842; *p* = 0.0671). The model had an R² of 0.2422 and a significant F value (F(2, 27) = 4.314; *p* = 0.0237).

### 3.7. Analysis of the Potential Effects of Confounding Factors on CMI

To investigate the effects of comorbidities presented by the volunteers as potential confounding factors, we applied a multiple linear regression model adjusted for CMI, with hypertension, Type 2 diabetes, cardiovascular disease, stroke, and cancer as the independent variables. According to this, none of the comorbidities showed a significant association with CMI, not only for the group with a normal weight (R² = 0.02936; F(5, 28) = 0.1694; *p* = 0.9718) but also for the group with obesity (R² = 0.1032; F(5, 24) = 0.5525; *p* = 0.7350), which indicated the very limited or even a residual influence of these variables on CMI.

## 4. Discussion

Overall, our data not only reinforce the observations that older adults with obesity present a more pronounced systemic pro-inflammatory status and CMI than older adults with adequate weight, but also, as a novelty of the study, demonstrate that the influence of the profile of circulating cytokines in CMI was found only in the group of older adults with an adequate weight.

As a pilot study, evaluating a wide range of cytokines with pro- and anti-inflammatory properties that are closely involved in the development and progression of inflammaging [10,11,12], as well as cardiometabolic and age-related diseases [23], can offer the opportunity to putatively establish which of them could be prominent candidates in the association between a systemic inflammatory status and CMI, particularly in older men.

According to the literature, significant elevations in levels of pro-inflammatory cytokines, such as IL-1β, IL-6, IL-12p70, IP-10, GM-CSF, TNF-α, and IFN-γ, as observed here in older men with obesity, agree with other studies that have documented similar increases in these pro-inflammatory cytokines in this population, indicating a close association between the state of chronic low-grade inflammation and obesity [24,25,26,27].

Clinically, the elevated systemic levels of IL-6 and TNF-α are particularly concerning, since these pro-inflammatory molecules are directly associated with insulin resistance and hyperinsulinemia, which are common in individuals with obesity. Although our sample did not show a significant difference in the incidence of Type 2 diabetes between the groups, these cytokines can still exacerbate the risk of developing this type of diabetes and cardiovascular diseases. For instance, IL-6 has been implicated in glucose metabolism and adipocytes’ function, contributing to insulin resistance and systemic inflammation [23,27], while the role of TNF-α in promoting insulin resistance is well documented [24].

Similarly, IL-1β is a pivotal mediator in inflammatory processes and has been shown to play a significant role in obesity-related inflammation, contributing to insulin resistance and β-cells’ dysfunction in diabetes [28]. Furthermore, IL-12p70, by promoting the differentiation of Th1 cells and stimulating the production of IFN-γ, can perpetuate the pro-inflammatory state, which may lead to chronic tissue damage and metabolic disturbances [22]. Additionally, elevated IFN-γ levels in individuals with obesity indicated that a Th1 immune response was elicited, which could be involved in the development and/or progression of chronic inflammation and autoimmunity. This cytokine is known to activate macrophages and enhance the production of other pro-inflammatory cytokines, thereby amplifying the inflammatory cascade. In addition, increased IFN-γ levels have been linked to metabolic syndrome and are thought to play a role in the development of insulin resistance and cardiovascular diseases [29,30]. Another cytokine with higher levels in individuals with obesity is GM-CSF, which plays a crucial role in the differentiation and activation of granulocytes and macrophages. 

Elevated GM-CSF levels can enhance the inflammatory response by promoting the survival and activation of these immune cells, which can contribute to chronic inflammation and tissue damage, in addition being involved in the pathogenesis of atherosclerosis and other inflammatory diseases [31]. In addition to these cytokines, individuals with obesity can also present higher circulating levels of IP-10 (also known an CXCL10), a chemokine involved in the recruitment of immune cells to sites of inflammation. On the basis of its action, it has been suggested that the increased chemotactic IP-10-derived activity in this population can drive the accumulation of immune cells in adipose tissue and other organs, exacerbating inflammation. Moreover, IP-10 is also associated with insulin resistance and has been found to correlate with the severity of metabolic and cardiovascular diseases [24]. Taken together, the reported information corroborates our findings and allows us to suggest that the elevated systemic pro-inflammatory status found here is likely a reflection of the compounding effects of age-related and obesity-related inflammation.

In an interesting way, the same group showed a significant increase in circulating levels of IL-10 as compared with the values found in the group with adequate weight, which could suggest that a regulated inflammatory state was present. However, the results obtained in the analysis of the ratio between the pro-inflammatory cytokines and the anti-inflammatory cytokine IL-10 may support the suggestion that volunteers with obesity presents a systemic pro-inflammatory status. At this point, it is of the utmost importance to point out that this analysis may provide an accurate measure regarding the inflammatory balance, which could help us to understand some adaptations or disturbances in older people [28,32,33]. 

Hence, the findings of the ratio-based analysis confirmed the presence of a systemic inflammatory imbalance in older adults with obesity, which could be associated with a disrupted immune response, since the regulatory mechanisms are insufficient to counteract the heightened inflammatory signals. It is also worth mentioning that studies have shown that this imbalance is a critical factor in the pathogenesis of obesity-related metabolic and cardiovascular diseases [24,26,34]. Furthermore, it was reported that older adults, even those of normal weight, are capable of exhibiting a pro-inflammatory bias, albeit to a lesser extent, compared with their counterparts with obesity [34].

In this context, Dragon-Durey et al. (2021) [35], Frasca et al. (2017) [36], and Shimi et al. (2024) [37] demonstrated that non-obese older adults can show a mild pro-inflammatory state characterized by elevated levels of cytokines, such as TNF-α, IL-6, and IL-1β, in conjunction with relative stability in the circulating levels of IL-10. Although this balance seems to be slightly skewed, it is generally able to maintain a minimum of immune regulation and homeostasis. However, as previously mentioned, in older adults with obesity, this balance is significantly disrupted, due to the combined effects of aging and obesity, which enhance a chronic low-grade inflammatory state. Therefore, the chronic maintenance of higher systemic levels of pro-inflammatory cytokines associated with an insufficient increase in IL-10 levels is considered to be a key driver of this imbalance [38].

Reinforcing the prominent differences in the systemic inflammatory state found here, the correlation analysis in the group with adequate weight showed moderate to strong associations, particularly with IL-10, which putatively indicated that a relatively balanced inflammatory status was achieved in these volunteers. In fact, this observation agrees with the literature, which demonstrates the maintenance of immune/inflammatory homeostasis, albeit slightly skewed, in older adults without obesity [39,40]. 

Conversely, the correlation analysis in the group with obesity showed a distinct profile among the cytokines assessed here, not only with fewer significant connections but also with lower values, which could putatively suggest a disturbance in the regulatory control of the inflammatory state [41,42,43]. Thus, the loss of this control, which leads to the development of a systemic profile towards an exacerbated pro-inflammatory state, has supposedly been linked to increased risks of metabolic and cardiovascular diseases, underscoring the importance of our findings [23].

In addition to the analysis of the inflammatory status, we also aimed to assess the interplay between this status and the CMI, since it is well documented that there is a close relation between inflammation and cardiometabolic health [42,43,44,45], Herein, the higher CMI values found in the group with obesity compared with the group with adequate weight corroborated the literature [14,46].

It is worth mentioning that the elevated CMI values observed in the group with obesity highlighted the presence of a substantial burden of cardiometabolic risk factors. The results obtained in the CMI-adjusted multivariate regression analysis showed a significant positive effect of IL-10 and a negative effect of IL-6 exclusively in the group with an adequate weight. These findings are very interesting and somewhat unexpected. Furthermore, they putatively indicate that a regulatory inflammatory profile, driven by IL-10 levels, does not directly affect the parameters involved in CMI, or it could represent an important player in the maintenance of cardiometabolic health [47], thus impacting the CMI.

Supporting this suggestion, the group with obesity did not show the significant effects of cytokines on CMI, possibly due to the prominent imbalance of the inflammatory state, which was mainly associated with the insufficient regulatory control of IL-10. Interestingly, it was reported that older adults with obesity who also had hypertension or dyslipidemia, but not diabetes, showed significant negative correlations between circulating IL-10 levels and alterations in the concentrations of total cholesterol, LDL-c, and HDL-c, but not with other cardiometabolic parameters, such as triglyceride levels, waist circumference, and BMI [47]. These data can help us to understand the lack of a significant effect of IL-10 on CMI in our group with obesity, since this cytokine did not show significant correlations with two important parameters involved in the CMI equation (triglycerides and waist circumference).

As far as we can understand, this is the first study that aimed to investigate the interplay between the levels of circulating cytokines and CMI in older men who presented with an adequate weight or obesity. Therefore, the study’s novelty was to show that the interplay between systemic inflammatory state and CMI can be influenced by the imbalance in this state towards a pro-inflammatory profile in older men.

Moreover, it is also important to mention that some aspects presented by the volunteers could represent confounding factors, which would lead to potential biases in this study, particularly aging-related diseases with a strong inflammatory component, such as hypertension, Type 2 diabetes, cardiovascular diseases, stroke, and cancer, as well as levels of physical activity. In order to mitigate these potential biases, firstly, we sought to homogenize the volunteer groups regarding the incidence of these comorbidities and the level of physical activity. In a general way, we achieved our goal, since the volunteer groups mostly had similar incidences of these comorbidities, except for hypertension, and the level of physical activity characterized the majority of volunteers in both groups as physically inactive (91.2% in the NW group and 96.9% in the O group). 

Furthermore, we also carried out a specific multiple regression analysis, which included the abovementioned confounding factors and was adjusted for CMI as the dependent variable, since this approach could improve our understanding of the effect of these comorbidities on CMI. Our results showed that none of these factors had a significant association with CMI, which could indicate a minimal or residual impact on the variability of CMI. Taken together, these findings allow us to putatively suggest that these confounding factors did not interfere with the regression models between the levels of circulating cytokines assessed here and CMI, and that the homogenization of confounding factors in the groups was satisfactory, demonstrating the robustness of our findings. Additionally, other potential confounding factors, such as dyslipidemia and the glycemic profile, did not show significant differences between the groups, showing that they also did not directly influence the results obtained when analyzing the inflammatory profile and its impact on CMI.

Beyond these pieces of information, it is worth mentioning that a systematic review reported that CMI is a useful tool to properly identify the risk of developing cardiometabolic disease in adults [48]; similarly, other studies have claimed its application in the older adult population [49,50,51]. Moreover, in terms of the immune/inflammatory aspects, it was also recently reported in the older adult population that all-cause mortality was positively associated with higher CMI, whereas a partial influence of some inflammatory parameters, particularly leukocytes (6.6%) and neutrophils (13.9%), was observed [52]. According to these data, whereas the use of CMI as a tool for the definition of several chronic and age-related diseases risk is increasing each day, the interplay of inflammatory parameters in the context of CMI, especially cytokines, is still unclear. Thus, our study can add an important piece to this puzzle and underscores the need for further studies to improve our understanding of how these pivotal elements—systemic inflammatory status and CMI—may be correlated and, subsequently, impact aging.

Lastly, beyond the significant findings presented here, our study has certain limitations. Firstly, although this was a pilot study and we reached the necessary number of participants to conduct it, the small number of volunteers in each group, combined with the number of variables assessed in the statistical analyses using multiple comparisons, may have led to the occurrence of Type I errors. However, this risk was mitigated by adjusting the α risk to 1% (*p* < 0.01) for the multivariate analyses, as described in the section on statistical analysis. Regardless, a higher number of volunteers would undoubtedly enhance the statistical robustness of our analysis and will be necessary to reproduce the findings of this study. Further limitations were the exclusive assessment of older men, as well as the fact that they were drawn entirely from a single region of Brazil; the lack of a comparison between our results and those of other groups, particularly younger people/adults with adequate weight or obesity; the lack of information on nutritional habits; and the inability to account for the continuous use of medications by the participants.

## 5. Conclusions

In the present study, it was reported for the first time that the regulated systemic inflammatory status observed in older people with an adequate weight, especially due to IL-10, had a significant impact on CMI, whilst the imbalance of this systemic inflammatory status towards a pronounced pro-inflammatory state profile, observed in the older people with obesity, led to the loss of any impact of this status on CMI. In addition, our findings reinforce the literature, since the elevated systemic pro-inflammatory status found in older men with obesity may not only be a reflection of the compounding effects of age-related and obesity-related inflammation but may also directly accelerate inflammaging.

## Figures and Tables

**Table 1:** Characteristics of the participants.

	NW	O	*p*-Value
**Demographic data**			
*n*	34	32	
Age	71.79 ± 6.85	71.78 ± 7.95	>0.05
**Anthropometric data**			
Height (cm)	168.80 ± 6.37	167.00 ± 7.54	>0.05
Weight (kg)	64.40 ± 6.50	93.35 ± 3.88	<0.0001
Waist circ. (cm)	87.72 ± 9.96	113.30 ± 22.99	<0.0001
BMI (kg/m²)	22.58 ± 1.53	33.31 ± 3.02	<0.0001
**Clinical data**			
HDL-c (mg/dL)	48.52 ± 9.70	37.15 ± 7.31	<0.0001
LDL-c (mg/dL)	117.4 ± 50.63	97.20 ± 30.74	>0.05
Triglycerides (mg/dL)	164.60 ± 48.84	200.80 ± 51.18	<0.001
Total cholesterol (mg/dL)	198.9 ± 59.78	164.2 ± 46.66	>0.05
Glucose (mg/dL)	104.7 ± 36.62	101.9 ± 36.63	>0.05
Hypertension	18 (52.9%)	30 (93.7%)	<0.001
DM-2	11 (32.3%)	17 (53.1%)	>0.05
CVD	8 (23.5%)	9 (28.1%)	>0.05
Stroke	2 (5.8%)	5 (15.6%)	>0.05
Cancer	4 (11.7%)	5 (15.6%)	>0.05
**Physical activity**			
Walking	13 (38.2%)	6 (18.7%)	>0.05
Light jogging	0	0	>0.05
Gymnastics	1 (2.9%)	0	>0.05
Weight training	0	1 (3.1%)	>0.05
Swimming	1 (2.9%)	1 (3.1%)	>0.05
Physically active	3 (8.8%)	1 (3.1%)	>0.05

NW, normal weight; O, obesity; HDL-c, high-density lipoprotein cholesterol; LDL-c, low-density lipoprotein cholesterol; DM-2, Type 2 diabetes mellitus; CVD, cardiovascular disease. Values are presented as the mean ± standard deviation.

## Data Availability

The data presented in this study are available on request from the corresponding author due to ethical reasons.
